# Supplementary material for: MS2Query: reliable and scalable MS2 mass spectra-based analogue search
Source: Nat Commun. 2023 Mar 29;14:1752. doi: 10.1038/s41467-023-37446-4 (PMC10060387; doi:10.1038/s41467-023-37446-4)
Supplement: Supplementary file 1 — Supplementary Information [file 41467_2023_37446_MOESM1_ESM.pdf]

Supplementary information with:

MS2Query: Reliable and Scalable MS^2 Mass Spectral-based Analogue Search

Niek F. de Jonge[1]*, Joris J. R. Louwen[1], Elena Chekmeneva[2], Stephane Camuzeaux[2], Femke J. Vermeir[3], Robert S. Jansen[3], Florian Huber[4]*, Justin J.J. van der Hooft [1,5]*

These authors jointly supervised this work: Florian Huber, Justin J.J. van der Hooft
*Corresponding authors: niek.dejonge@wur.nl, florian.huber@hs-duesseldorf.de, justin.vanderhooft@wur.nl

Affiliations:

1 Bioinformatics Group, Wageningen University, 6708 PB Wageningen, the Netherlands
2 National Phenome Centre, Section of Bioanalytical Chemistry, Division of Systems Medicine, Department of Metabolism, Digestion and Reproduction, Faculty of Medicine, Imperial College London, Hammersmith Hospital Campus, London, W12 0NN, United Kingdom
3 Department of Microbiology, Radboud Institute for Biological and Environmental Sciences, Radboud University, 6525ED Nijmegen, the Netherlands
4 Centre for Digitalization and Digitality (ZDD), University of Applied Sciences Düsseldorf, Düsseldorf, Germany
5 Department of Biochemistry, University of Johannesburg, Auckland Park, Johannesburg 2006, South Africa

Supplementary Note 1: Model used for Case studies

The model used for the optimization of the hyperparameters and for the case studies was trained on positive ionization mode mass spectra from GNPS (https://gnps-external.ucsd.edu/gnpslibrary/ALL_GNPS) downloaded on the 15th of November 2021, 20:00 CET. The version of MS2Query used for the training of this model was version 0.3.2, the main difference with this version is that the training of the models was done in notebooks, instead of having a fully automatic pipeline.

The same training workflow was used as for the k-fold cross-validation except for the sizes of the test sets and validation set split used. The "analogue test set" for this model was generated by randomly selecting 250 unique 2D molecules from the library and selecting all corresponding spectra. The "exact matches test set" for this model was generated by randomly selecting 3000 spectra from the library with at least one 2D structure match in the training spectra.

In addition, a validation test set was used. This was used for optimizing hyperparameters of MS2Query and to decide which features to include in the Random Forest model. The validation test set for MS2Query consisted of all spectra of 50 unique 2D structures and 600 spectra with at least one 2D structure match in the training spectra.

The model was trained in the same way as the k-fold validation but the number of training pairs was slightly different. For generating the training spectrum pairs for the random forest model of MS2Query a set of spectra was used containing all spectra of 200 unique 2D structures and 2400 spectra with at least one 2D structure match in the training spectra.

When aiming for a recall of 35%, the MS2Query threshold for this test set is 0.633 and results in an average Tanimoto score of 0.67 (Supplementary Figure 3 shows a detailed Tanimoto score distribution).

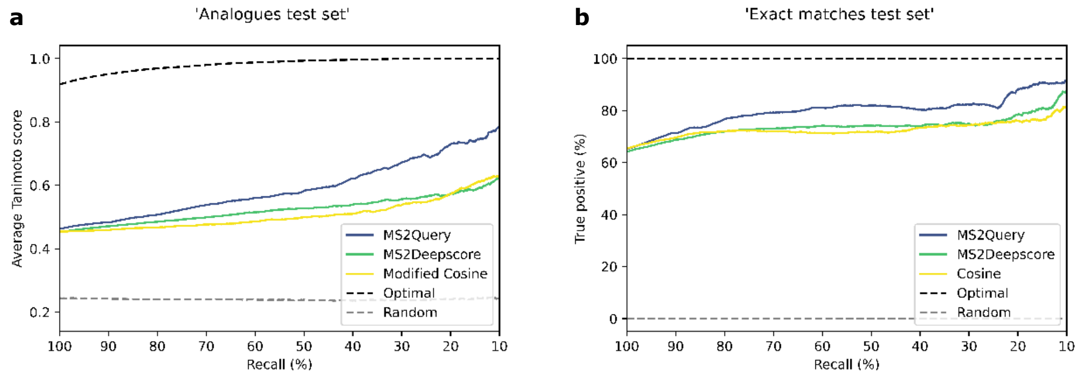

Supplementary Figure 1: Performance of the model used for the case studies. Source data are provided as a Source Data file. ***A:*** *The 'analogues test set' is used with spectra that have no exact match in the library, therefore the best possible match is always an analogue. For MS2Deepscore, cosine score and modified cosine score, library spectra are first filtered on a mass difference of 100 Da. The relationship between recall and average Tanimoto score (chemical similarity) is plotted. For each threshold the average over the Tanimoto scores between the correct molecular structure and the predicted analogues is calculated.* ***B:*** *The 'exact match test set' is used, all these test spectra have at least 1 exact structural match in the reference library. For MS2Deepscore and modified cosine score, library spectra are first filtered on a mass difference of 0.25 Da, while MS2Query does not use any pre-filtering on mass difference, and uses the exact same settings as for the analogue search. The percentage of true positives is given. A match is marked as true positive if the 2D structure is correct.*

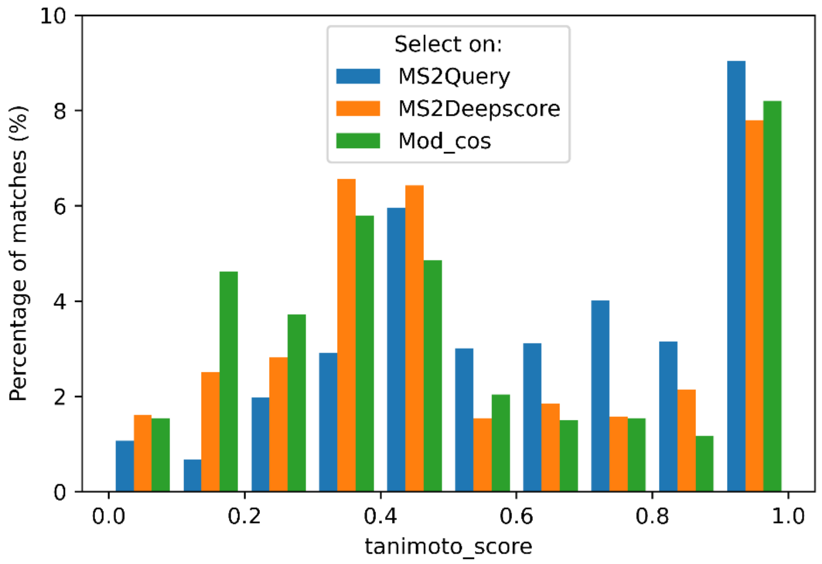

Supplementary Figure 2: The distribution of Tanimoto scores between the correct match and the best found match. The "Analogues test set" is used with spectra that have no exact match in the library, therefore the best possible match is always an analogue. The minimal threshold for MS2Query, MS2Deepscore and Modified cosine is set to result in a recall of 35% for this test set. A histogram is plotted to show the number of spectra in each subset. Source data are provided as a Source Data file.

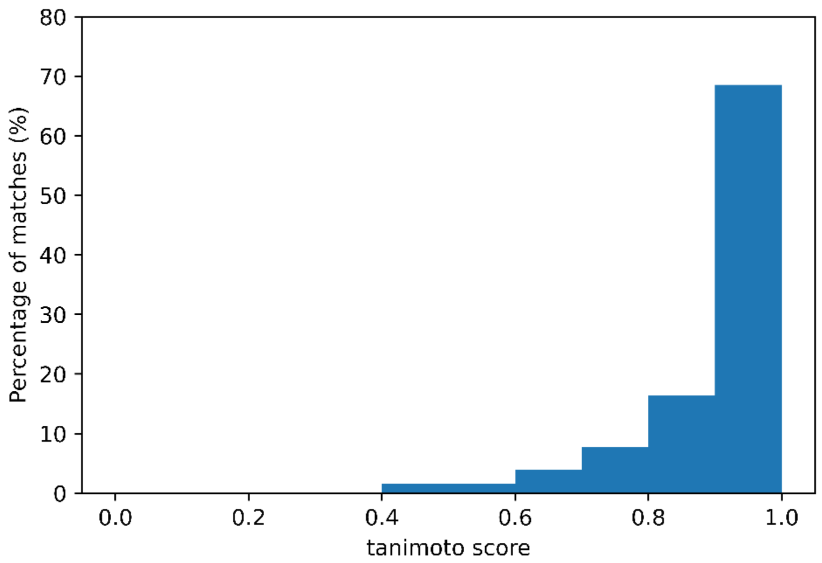

Supplementary Figure 3: Distribution of Tanimoto scores between the true structure and the best possible match in the reference library for the "Analogues test set". Source data are provided as a Source Data file.

Supplementary Note 2: Feature importance

The resulting mean squared error (MSE) for the MS2Query random forest model predicting Tanimoto scores is 0.0282 for the training data and 0.0255 for the validation data. The feature importance of the different features is given in Table S1. This method is based on an impurity-based feature importance, also known as the Gini importance[1]. As an alternative method for assessing the impact the features have on the model performance, random forest models were trained lacking one of the features, to determine the difference in performance for these models (Table S2). Replacing the average MS2Deepscore of multiple library structures with the MS2Deepscore between 1 library spectrum and the query spectrum increased the MSE from 0.0255 to 0.0337 for the validation data set. This clearly demonstrates that using the average of multiple similar library spectra significantly improves the performance of the random forest model. Removing any of the other features also decreased the performance of the model.

Supplementary Table 1: Feature importance for the 5 features used by the random forest.

**Feature**	**Feature Importance**
Average of MS2Deepscore of multiple similar library spectra	0.62
Precursor m/z difference	0.18
Query spectrum m/z	0.13
Spec2Vec score	0.05
Average Tanimoto score for similar library spectra	0.02

Supplementary Table 2: The effect on the MSE, when the random forest model is trained without one of the features.

**Removed feature**	**Training MSE**	**Validation MSE**
No feature removed	0.0282	0.0255
Average MS2Deepscore of multiple library structures	0.0339	0.0331
Precursor m/z difference	0.0311	0.0283
Query precursor m/z	0.0300	0.0265
Spec2Vec	0.0290	0.0272
Average Tanimoto score of similar library structures	0.0283	0.0259

Rationale behind precursor m/z difference

One of the newly introduced feature of MS2Query is using precursor m/z difference as an input feature for the random forest model. Current implementations of an analogue search often start with a preselection on precursor m/z followed by selecting spectra above a predefined threshold for the used similarity score[2]. A predefined precursor m/z threshold does not differentiate between precursor m/z differences as long as the mass difference falls within the set threshold. However, certain specific mass differences will be more likely than others. By using the precursor m/z difference as an input feature, the random forest can learn specific mass differences that are more likely to correspond to a good analogue or exact match. This approach has similarities to the analogue search implementation by Stephen E. Stein and colleagues: in their approach, they limit the analogues to a predefined list of precursor m/z differences of commonly observed losses[3]. This is an interesting approach; however, this limits the analogues only to known losses and does not allow for analogues that have a substructure substituted by another substructure. Our random forest model is more flexible and can learn the mass differences that are an indication of a good analogue, even if these are not known mass differences.

Supplementary Note 3: Testing additional features for MS2Query random forest model

Five features were used as input for the current random forest model. However, multiple other features and variations of the current features were tested to select the features that contain information to predict chemical similarity. The performance of multiple models with different feature sets is compared. The feature importance is calculated for each model and used as a first guide for selecting important or relevant features, followed by training a new model with the selected features, to make sure the performance does increase. The performance of a model is measured by the mean squared error for the training and validation dataset. The code used to calculate these other features can be found in the branch https://github.com/iomega/ms2query/tree/add_cosine_to_features on the MS2Query Github repository, the notebooks for the generation of the random forest models can be found on https://github.com/iomega/ms2query/blob/add_cosine_to_features/notebooks/Analysis_with_dataset_GNPS_15_12_2021/test_features_for_RF/test_random_forest_with_49_features.ipynb. Below, alternative possible MS2Query input features are discussed in detail.

Modified cosine score and cosine score

Both cosine scores and modified cosine scores were added as features for training random forest models to explore if they would notably contribute to the model predictions. However, the feature importance was always 0 for these scores, indicating that the model does not use the (modified) cosine score for the prediction at the set tree depth of 5. This suggests that the (modified) cosine score does not provide additional predictive power when MS2Deepscore and Spec2Vec are part of the model as well. Therefore, it was decided to not incorporate the (modified) cosine score in the workflow of MS2Query.

Weighting of the average of multiple library structures

The multiple library spectra were selected from the 10 library structures that are chemically most similar to the structure of interest, based on the Tanimoto scores. For all spectra belonging to these 10 selected library spectra, we compute the MS2Deepscore towards the query spectrum. To find a good, reliable method for taking an average and for weighting each of these selected spectra, we tried different approaches and selected the method with the best performance. To assess which method works best the MSE was compared for two models having different approaches for this feature, while the other 4 features stayed constant (precursor m/z difference, query precursor m/z, spec2vec_score, and MS2Deepscore).

There are two approaches that we assessed for calculating the average of the calculated MS2Deepscores. The first method just takes the average over all selected spectra regardless of which library structure it belongs to and the second method first calculates the average MS2Deepscore for each selected library structure, followed by taking the average of the 10 average scores for each library structure. Since for

one InChIKey the number of library spectra differs, the two methods result in a different way of weighting the spectra.

The model using the feature that first calculates the average MS2Deepscore for each selected library structure, followed by taking the average of the 10 average scores for each library structure performed the best. This method had an MSE of 0.0283 for the training set and 0.0257 for the validation set. The model using the feature where the average is just taken over all spectra had an MSE of 0.0301 for the training data and an MSE for the validation data of 0.0286. Therefore, the first method of selecting the average was selected.

Weighting based on Tanimoto score

In the comparison done in S2.2 each library structure is weighted equally. In practice, however, we see variations in how chemically similar the 10 closest structures are (all measured using Tanimoto). To explore if this has any notable negative impact on our model performance, we tested a weighted average. This should give more importance to library molecules that are more similar. We hence weighted each score by the Tanimoto score and further also tested different powers of this weighting. A higher power will result in more extreme weight to spectra of more similar library structures and a low power will have less effect.

As weight, the Tanimoto score to the power of 1-3 was tested. In Table S3 the performance of the different models are shown. This shows that using weighting of the different structures based on the Tanimoto score did not improve the overall performance of the model. Therefore, no weighting is used for calculating the feature using multiple library structures.

Supplementary Table 3: Training and validation MSE for different weighting methods for the average MS2Deepscore of multiple library structures.

Method of weighting	Training MSE	Validation MSE
No weighting	0.0283	0.0257
Tanimoto score	0.0284	0.0260
Tanimoto score^2	0.0286	0.0262
Tanimoto score^3	0.0290	0.0265

Mass spectrometer instrument type

The mass spectra in the GNPS library are measured on a wide variety of mass spectra instruments. We tested if we could use this information to improve the performance of the random forest model, since it is conceivable that some features are influenced by the instrument type or by whether or not the two spectra of interest were measured on different instruments. To this end we used metadata of spectra to classify the query and library spectra as a mass spectrometer using "ToF", "Quadrupole", "Ion Trap", or "Orbitrap" setup. Instruments that were not given or used a system that could not be classified as one of the 4 mentioned techniques were not classified. For both the query spectrum and the library spectrum a binary feature is created for each of these mass spectrum instrument types. Thus, this resulted in 4 features to indicate the type of the query spectrum and 4 features to indicate the type of the library spectrum: a total of 8 features. The value was set to 1 if it was measured on this instrument type and 0 if it was not measured on this instrument type. If the instrument type could not be classified, all features were set to 0.

The feature importance of training a random forest model including these features always resulted in a feature importance of 0. This shows that with this setup, the random forest model was not able to use

this information to increase the prediction quality of MS2Query. Therefore, this feature was left out in the current MS2Query workflow.

Supplementary Note 4: Performance of an analogue search for different mass ranges

increased performance for larger metabolites

Many tools for MS^2 spectrum annotation do not perform equally well for low and high query masses[4-6]. For MS2Query the performance for different masses is tested by splitting the 'analogues test set' with spectra without an exact match in the library into three mass ranges; 0-300 Da, 300-600 Da and > 600 Da. Figure 3 displays the Tanimoto score distributions of the suggested analogues for these three mass ranges. This analysis reveals that MS2Query performs best for large metabolites (>600 Da) where it detected analogues with an average Tanimoto score of 0.85 and has a recall of 63%. A better performance for larger metabolites can also be observed when using MS2Deepscore, cosine or modified cosine score. The plots showing optimal and random results show the same results, suggesting that at least part of this improved performance for larger masses is an artifact of the spectral libraries or the Tanimoto score. However, the increase for the large masses seems to be more pronounced for the analogue search methods compared to the random search. A possible explanation why analogue searching is more accurate for larger metabolites, is that larger metabolites will often produce a higher number of characteristic fragments. In practice, the observed high analogue similarity for larger molecules is interesting, since it is complementary to currently existing methods relying on fragmentation tree-based approaches. Fragmentation tree-based methods perform well for smaller metabolites <500 Da, but perform less well for larger metabolites, both in terms of computational time and reliability[4, 6, 7]. This shows the potential for combining the two approaches and using the best of both for optimal performance.

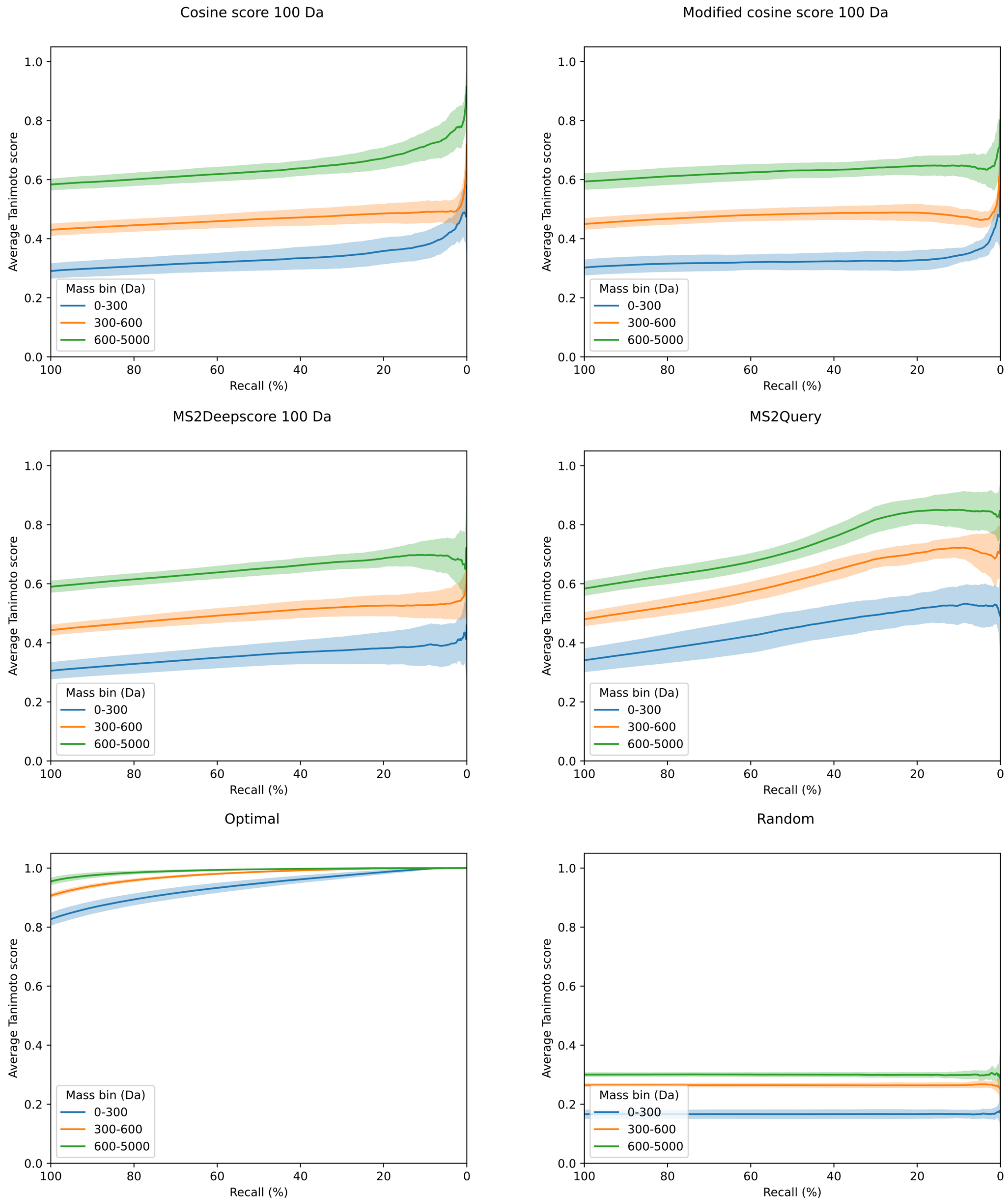

Supplementary Figure 4: Recall vs quality plots, with test sets split on mass bins. These are the same test sets used for the "analogue test set" in the k-fold cross-validation, but here each test set is split on the precursor m/z of the test spectrum. The mean of these 20 test sets are shown and the standard deviation is highlighted. Source data are provided as a Source Data file.

Supplementary Note 5: Justification for method of selecting training data

Pairs of spectra were used for the training data of the random forest. If the spectra pairs would have been picked at random, the resulting Tanimoto scores would be very low on average[8]. To prevent overfitting to low Tanimoto scores, it is important that a more equal distribution of Tanimoto scores is used for training. To achieve this, the spectrum pairs were picked by selecting the top 100 highest scoring library spectra for MS2Deepscore for a set of training spectra. This method for selecting spectrum pairs of spectra for training is similar to the workflow for running MS2Query and results in a relatively equal distribution of Tanimoto scores between these spectra, preventing a bias towards predicting lower Tanimoto scores.

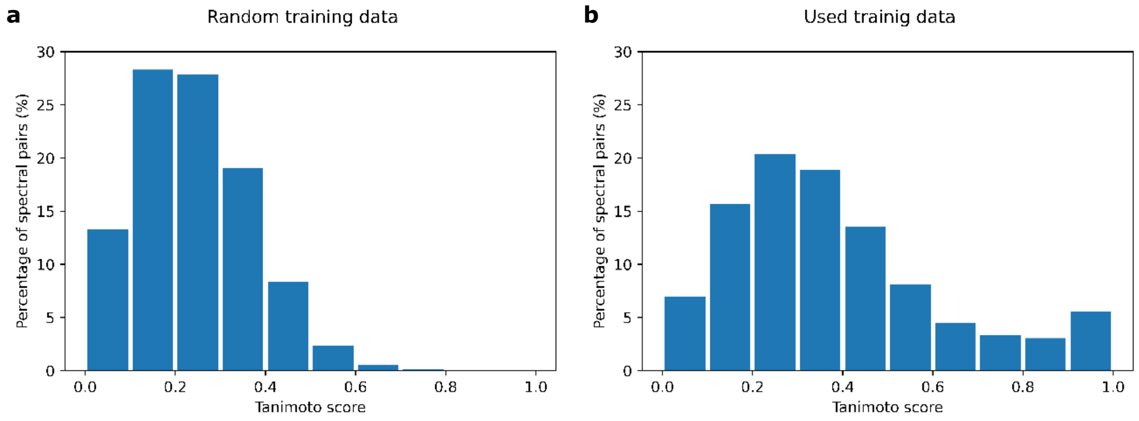

Supplementary Figure 5: Comparison of the distribution of Tanimoto scores between spectral pairs in the library and the distribution for the used training data. Source data are provided as a Source Data file. ***a****: Distribution of Tanimoto scores when selecting random spectral pairs. Spectral pairs are selected by making a pair between the training spectra and all InChiKeys in the library.* ***b****: Distribution of Tanimoto scores between the pairs of spectra used for training the random forest model. These spectral pairs were selected by calculating MS2Deepscore between each training spectrum and the reference library and making pairs with the 100 highest scoring library spectra.*

Supplementary Note 6: Additional analysis case study GNPS

The GNPS analogue search was run for case studies 1-3 using a mass window of 200 Da, a cosine score threshold of 0.6, minimum matched peaks set to 3, the ion mass tolerance set to 0.25 Da and a fragment Ion Mass Tolerance of 0.01 Da.

A molecular network was created with the Feature-Based Molecular Networking (FBMN) workflow (Nothias et al., 2020) on GNPS (https://gnps.ucsd.edu, (Wang et al., 2016). The feature quantification table and MS2 spectral summary file were uploaded to GNPS. The data were filtered by removing all MS^2 fragment ions within +/- 17 Da of the precursor m/z. MS^2 spectra were window filtered by choosing only the top 6 fragment ions in the +/- 50 Da window throughout the spectrum. The precursor ion mass tolerance was set to 0.02 Da and the MS^2 fragment ion tolerance to 0.02 Da. A molecular network was then created where edges were filtered to have a cosine score above 0.7 and more than 3 matched peaks. Further, edges between two nodes were kept in the network if and only if each of the nodes appeared in each other's respective top 10 most similar nodes. Finally, the maximum size of a molecular family was set to 100, and the lowest scoring edges were removed from molecular families until the molecular family size was below this threshold.

The jobs can be publicly accessed at:

Blood plasma LTR analogue search:
https://gnps.ucsd.edu/ProteoSAFe/status.jsp?task=5d4577850dae44758da85c6ee3b77e89

Urine LTR analogue search:
https://gnps.ucsd.edu/ProteoSAFe/status.jsp?task=303ee7013d994074b27de8b86fd3bbad

Blood plasma NIST 1950 analogue search:
https://gnps.ucsd.edu/ProteoSAFe/status.jsp?task=6f0c60c689bb4facb64e96fac968ff0d

Anammox bacteria molecular networking:
https://gnps.ucsd.edu/ProteoSAFe/status.jsp?task=250044393bf44654ad72b7cebebba478

The results of the analogue searches are merged with the annotated csv files. Despite our efforts, it is challenging to judge for a case study what method performs better, due to the relatively low number of (tentatively) validated spectra and the fact that judging the quality of analogues is somewhat subjective. However, for case study 1 a relatively high number of case study spectra could be validated. In total, for 67 spectra, the precursor m/z and retention time could be linked to an in-house reference. Both the results predicted by GNPS and MS2Query were compared to the reference standards. The biggest difference was in the spectra that we were not able to validate, which makes it challenging to directly compare the performance of GNPS analogue search with GNPS for this case study.

Supplementary Table 4: Results of manual validation of results of MS2Query and GNPS analogue search for the NIST blood plasma case study.

	MS2Query	GNPS
Correct	9	6
Good analogue	31	40
Analogue	16	11
Bad analogue	4	4
Wrong	1	0
Unknown	14	29
Unannotated	28	13
Total	103	103

Supplementary Note 7: Performance without precursor m/z preselection

Benchmarking with Cosine score, Modified cosine score and MS2Deepscore was done with a preselection on mass difference between a query and library molecule of 100 Da. In Supplementary Figure 7, we compare the difference in performance with and without a preselection on mass difference. These results show that performance is slightly better when using prefiltering on a mass difference of 100 Da, when using MS2Deepscore and the cosine score. When using the modified cosine score the results are a bit better for low recall settings, when no preselection on mass difference is done, however this comes at the cost of a large increase in runtime.

In addition a new MS2Deepscore model was trained that already includes the precursor m/z during training. The performance of this model is slightly better, but still MS2Query performs better.

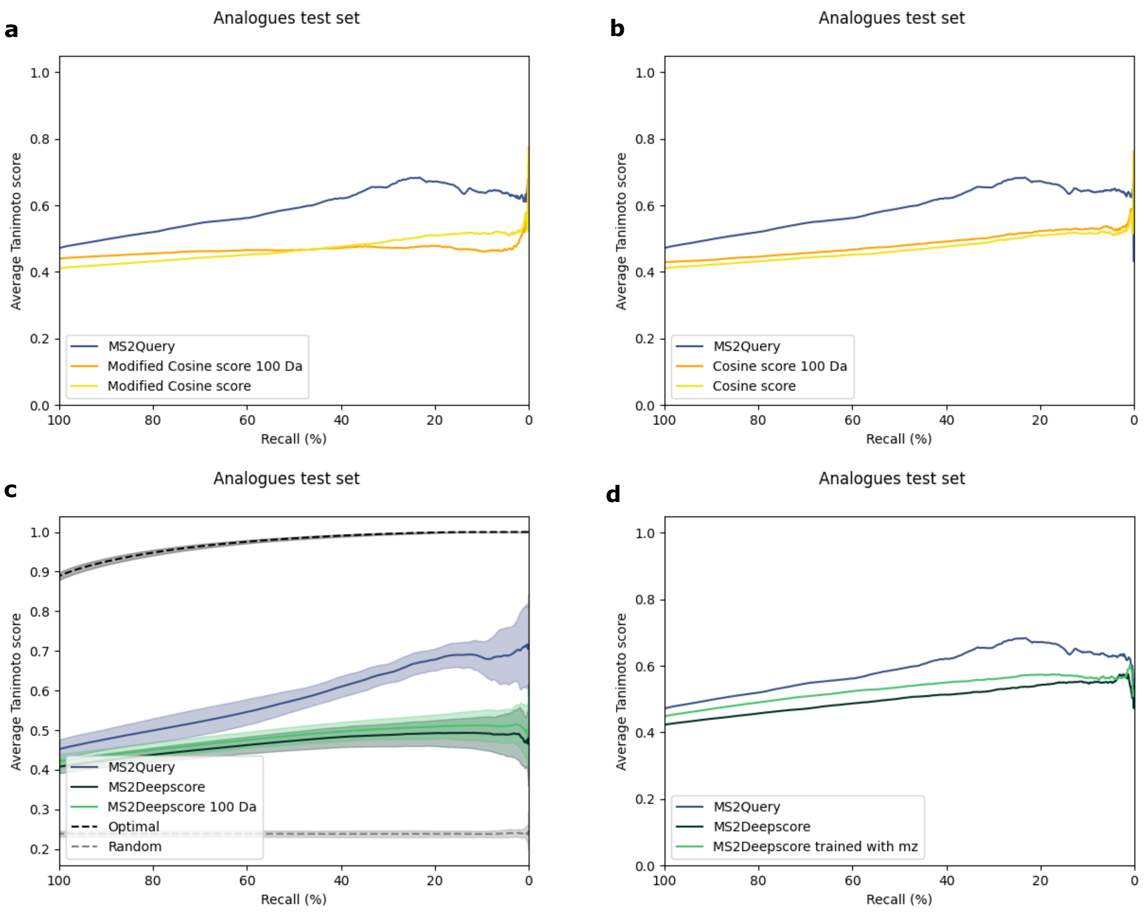

Supplementary Figure 6: Comparisons of performance for variants of the reference benchmarking used in Figure 2. Source data are provided as a Source Data file. ***a:*** *Modified Cosine score with a preselection on a mass difference of 100 Da performs slightly better at low recall. The first test set of the 20-fold cross-validation was used.* ***b:*** *Cosine score with a preselection on a mass difference of 100 Da performs slightly better than the cosine score without any preselection on mass difference. The first test set of the 20-fold cross-validation was used.* ***c:*** *MS2Deepscore with preselection on a mass difference of 100 Da performs slightly better than MS2Deepscore without any preselection on mass difference. The same 20-fold cross-validation test sets are used as in the main text. The mean of these 20 test sets are shown and the standard deviation is highlighted.* ***d:*** *MS2Deepscore model trained including precursor m/z. This model performs slightly better than the MS2Deepscore model trained without this feature, however MS2Query still performs better compared to this model. The first test set of the 20-fold cross-validation was used.*

Supplementary Note 8: Cosine score and Modified Cosine score

The cosine score and modified cosine score are used as reference benchmarking. The Cosine score and modified cosine score both calculate spectral similarity by directly comparing spectral peaks. The score is calculated by finding the best possible matches between peaks of two spectra and subsequently calculating a cosine score between the resulting vectors.

For the comparison of a spectrum S to another spectrum S' a peak p is considered as an eligible match for the cosine score if $mz(p)-mz(p') < t$, where t is the tolerance. The tolerance used was 0.05 Da.

For the modified cosine score a peak is also considered as an eligible match if $mz(p)-mz(p') < t$, but also if $mz(p) + M - mz(p') < t$, where $M=PM(S')-PM(S)$. M is the modification mass and PM is the Precursor mass.

Supplementary Note 9: Methods details listed in Tables

Supplementary Table 5: Lipid standard mixture.

Lipid	Stock conc. (mg/mL)	Conc. (µg/mL) in Lipid Mix
LPC(9:0)	0.5	0.25
PC(11:0/11:0)	0.1	0.25
FA(17:0)	0.1	2.50
PG(15:0/15:0)	0.01	1.00
PE(15:0/15:0)	0.01	0.25
PS(17:0/17:0)	0.1	6.00
PA(17:0/17:0)	0.1	1.00
Cer(d18:1/17:0)	0.1	0.05
DG(19:0/19:0)	0.2	12.00
PC(23:0/23:0)	0.1	0.25
TG(15:0/15:0/15:0)	0.1	2.50
TG(17:0/17:0/17:0)	0.1	2.50

Supplementary Table 6: HILIC MR and IS mixture

**Compound**	**MR/IS**	**Conc. in MR or IS solution (µM)**
L-Phenylalanine-$^{13}C,^{15}N_9$	MR	2400
Adenine-2d1	MR	192
Taurine-^{15}N	MR	2400
Creatine-d3 H_2O	MR	240
L-Arginine-$^{13}C_6$	MR	2400
L-Tryptophan-d5	MR	2400
Uracil-2-$^{13}C,^{15}N_2$	MR	2400
N-Benzoyl-d_5-glycine	IS	4800
Adenosine-2-d-1	IS	384

Supplementary Table 7. RP MR and IS mixture

Compound	MR/IS	Conc. in MR or IS solution (**µM)**
L-Glutamic Acid-$^{13}C_5$	MR	2000
L-Isoleucine-$^{13}C_6$,^{15}N	MR	7500
L-Leucine-$^{13}C_6$	MR	7500
L-Tryptophan-$^{13}C_{11}$,$^{15}N_2$	MR	1500
Octanoic Acid-$^{13}C_8$	MR	1000
L-Glutamine-$^{13}C_5$	MR	12500
Creatinine-Methyl-d_3	MR	10000
Cytidine-5,6-d_2	MR	4000
Citric Acid-$^{13}C_6$	MR	5000
Benzoic Acid-Ring-$^{13}C_6$	MR	2000
L-Phenylalanine-$^{13}C_9$,^{15}N	IS	600
(N-Benzoyl-d_5-Glycine) Hippuric Acid-d_5	IS	500

Supplementary Table 8. RPC lipid profiling LC gradient elution program

LC Gradient					
**#**	**Time (min)**	**Flow (mL/min)**	**% A**	**% B**	**Curve**
1	Initial	0.6	99.0	1.0	Initial
2	0.10	0.6	99.0	1.0	6
3	2.00	0.6	70.0	30.0	6
4	11.50	0.6	10.0	90.0	6
5	12.00	1.0	0.1	99.9	6
6	12.50	1.0	0.1	99.9	6
7	12.55	0.9	35.0	65.0	6
8	12.65	0.8	70.0	30.0	6
9	12.75	0.7	99.0	1.0	6
10	12.95	0.6	99.0	1.0	6
11	13.25	0.6	99.0	1.0	6

Supplementary Table 9. HILIC profiling LC gradient elution program

Gradient					
**#**	**Time (Mins)**	**Flow (ml/min)**	**% A**	**% B**	**Curve**
1	Initial	0.600	5.0	95.0	Initial
2	0.1	0.600	5.0	95.0	6
3	4.6	0.600	20.0	80.0	6
4	5.5	0.600	50.0	50.0	6
5	7.0	0.600	50.0	50.0	6
6	7.1	0.605	5.0	95.0	6
7	7.2	0.610	5.0	95.0	6
8	7.3	0.620	5.0	95.0	6
9	7.4	0.650	5.0	95.0	6
10	7.5	0.700	5.0	95.0	6
11	7.6	0.800	5.0	95.0	6
12	7.7	0.900	5.0	95.0	6
13	7.8	1.000	5.0	95.0	6
14	12.50	1.000	5.0	95.0	6
15	12.65	0.600	5.0	95.0	6

Supplementary Table 10. RPC urine profiling LC gradient elution program

Gradient					
**#**	**Time (Mins)**	**Flow (ml/min)**	**% A**	**% B**	**Curve**
1	Initial	0.600	99.0	1.0	Initial
2	0.10	0.600	99.0	1.0	6
3	10.00	0.600	45.0	55.0	6
4	10.15	0.610	35.0	65.0	6
5	10.30	0.630	25.0	75.0	6
6	10.45	0.670	15.0	85.0	6
7	10.60	0.750	5.0	95.0	6
8	10.70	0.800	0.0	100.0	6
9	11.00	1.000	0.0	100.0	6
10	11.55	1.000	0.0	100.0	6
11	11.65	1.000	99.0	1.0	6
12	11.70	0.900	99.0	1.0	6
13	11.80	0.800	99.0	1.0	6
14	11.90	0.700	99.0	1.0	6
15	12.00	0.650	99.0	1.0	6
16	12.10	0.610	99.0	1.0	6
17	12.15	0.600	99.0	1.0	6
18	12.65	0.600	99.0	1.0	6

Supplementary References

1. Breiman, L., Friedman, J.H., Olshen, R.A. & Stone, C.J. Classification and regression trees. (Routledge, 2017).
2. Wang, M. et al. Mass spectrometry searches using MASST. *Nature biotechnology* **38**, 23-26 (2020).
3. Cooper, B.T. et al. Hybrid search: a method for identifying metabolites absent from tandem mass spectrometry libraries. *Analytical chemistry* **91**, 13924-13932 (2019).
4. de Jonge, N.F. et al. Good Practices and Recommendations for Using and Benchmarking Computational Metabolomics Metabolite Annotation Tools. (2022).
5. Huber, F. et al. Spec2Vec: Improved mass spectral similarity scoring through learning of structural relationships. *PLoS Comput. Biol.* **17**, e1008724 (2021).
6. Böcker, S. & Dührkop, K. Fragmentation trees reloaded. *J. Cheminform.* **8**, 5 (2016).
7. Böcker, S., Letzel, M.C., Lipták, Z. & Pervukhin, A. SIRIUS: decomposing isotope patterns for metabolite identification†. *Bioinformatics* **25**, 218-224 (2008).
8. Huber, F., van der Burg, S., van der Hooft, J.J.J. & Ridder, L. MS2DeepScore: a novel deep learning similarity measure to compare tandem mass spectra. *J. Cheminform.* **13**, 84 (2021).
