# Supplementary material for: MS2Query: reliable and scalable MS2 mass spectra-based analogue search
Source: Nat Commun. 2023 Mar 29;14:1752. doi: 10.1038/s41467-023-37446-4 (PMC10060387; doi:10.1038/s41467-023-37446-4)
Supplement: Supplementary file 3 — Reporting Summary [file 41467_2023_37446_MOESM3_ESM.pdf]

nature portfolio

Corresponding author(s): Niek F. de Jonge, Florian Huber, Justin J.J. van der Hooft

Last updated by author(s): 03-03-2023

Reporting Summary

Nature Portfolio wishes to improve the reproducibility of the work that we publish. This form provides structure for consistency and transparency in reporting. For further information on Nature Portfolio policies, see our Editorial Policies and the Editorial Policy Checklist.

Statistics

For all statistical analyses, confirm that the following items are present in the figure legend, table legend, main text, or Methods section.

n/a	Confirmed	
x	☐	The exact sample size (*n*) for each experimental group/condition, given as a discrete number and unit of measurement
x	☐	A statement on whether measurements were taken from distinct samples or whether the same sample was measured repeatedly
x	☐	The statistical test(s) used AND whether they are one- or two-sided<br>*Only common tests should be described solely by name; describe more complex techniques in the Methods section.*
x	☐	A description of all covariates tested
x	☐	A description of any assumptions or corrections, such as tests of normality and adjustment for multiple comparisons
x	☐	A full description of the statistical parameters including central tendency (e.g. means) or other basic estimates (e.g. regression coefficient) AND variation (e.g. standard deviation) or associated estimates of uncertainty (e.g. confidence intervals)
x	☐	For null hypothesis testing, the test statistic (e.g. *F*, *t*, *r*) with confidence intervals, effect sizes, degrees of freedom and *P* value noted<br>*Give P values as exact values whenever suitable.*
x	☐	For Bayesian analysis, information on the choice of priors and Markov chain Monte Carlo settings
x	☐	For hierarchical and complex designs, identification of the appropriate level for tests and full reporting of outcomes
x	☐	Estimates of effect sizes (e.g. Cohen's *d*, Pearson's *r*), indicating how they were calculated

Our web collection on statistics for biologists contains articles on many of the points above.

Software and code

Policy information about availability of computer code

Data collection	Data collection was performed using Agilent Masshunter software 10.0 (Agilent Technologies).
Data analysis	The data analysis was done using MS2Query. The case study results were obtained using version 0.3.2 of MS2Query and the k-fold cross-validation results were obtained using version 0.5.6. MS2Query has dependencies on other packages that can be found in the setup.py. The versions of these dependencies used for generating the results are matchms=0.13.0, numpy=1.21.6, spec2vec=0.6.0, tensorflow=2.8.0, scikit-learn=0.24.2, ms2deepscore=0.3.0, gensim=4.2.0, pandas=1.3.5, matchms2xtras=0.3.0, pubchempy=1.0.4, tqdm=4.64.0, matplotlib=3.5.1. When installing ms2query via pip install, these packages will be automatically installed as well.

For manuscripts utilizing custom algorithms or software that are central to the research but not yet described in published literature, software must be made available to editors and reviewers. We strongly encourage code deposition in a community repository (e.g. GitHub). See the Nature Portfolio guidelines for submitting code & software for further information.

Data

Policy information about availability of data

All manuscripts must include a data availability statement. This statement should provide the following information, where applicable:

- Accession codes, unique identifiers, or web links for publicly available datasets
- A description of any restrictions on data availability
- For clinical datasets or third party data, please ensure that the statement adheres to our policy

The models and spectra files used for the case studies can be downloaded from https://zenodo.org/record/6124553. For the k-fold cross validation the raw results,

the raw data and the data splits can be downloaded from https://zenodo.org/record/7427094.
The mass spectrometry data for the case studies were deposited in the MassIVE repository (accession number MSV000089648 and MSV000090642).
For validation of the case study results multiple libraries were used: LIPID MAPS https://www.lipidmaps.org/, HMDB https://hmdb.ca/ and GNPS https://gnps.ucsd.edu/.

Human research participants

Policy information about studies involving human research participants and Sex and Gender in Research.

Reporting on sex and gender	A Long-Term Reference (LTR) urine sample was created by pooling seventy-eight individual urine voids collected in a single day from volunteer subjects. No data about sexes was collected.
Population characteristics	No screening criteria were used to assess the health status of the donors.
Recruitment	Recruitment was on voluntary basis. This can cause bias in the sense that it does not represent an average urine sample, this is not expected to impact the results of this study, since the case study is used to illustrate the performance of MS2Query on real samples, not to discuss the average contents of urine.
Ethics oversight	LTR urine collection was carried out under REC Wales approval: 12/WA/0196.

Note that full information on the approval of the study protocol must also be provided in the manuscript.

Field-specific reporting

Please select the one below that is the best fit for your research. If you are not sure, read the appropriate sections before making your selection.

☒ Life sciences ☐ Behavioural & social sciences ☐ Ecological, evolutionary & environmental sciences

For a reference copy of the document with all sections, see nature.com/documents/nr-reporting-summary-flat.pdf

Life sciences study design

All studies must disclose on these points even when the disclosure is negative.

Sample size	For benchmarking MS2Query, all publicly available data on GNPS was used, containing over 300,000 mass spectra at the time of analysis, to start with. To do the benchmarking, after selecting based on quality of mass spectra and metadata (see Data exclusions), the dataset was split into 20 different sets that were the input for 20-fold cross-validation.
Data exclusions	Mass spectra containing not sufficient mass peaks after noise filtering steps (see Methods section) were excluded. Mass spectra for which no full molecule annotation by the means of Inchi, InchiKey, and/or SMILES was available were also excluded from the benchmarking since the accuracy of predictions cannot be validated fort these entries.
Replication	By doing 20-fold cross-validation we tested the replicability of the study. By training 20 different machine learning models and using 20 different randomized test sets we showed that the performance generalizes for different test sets and training data. There are different outcomes for the different models and test sets, but these differences do not change the general conclusions.
Randomization	The allocation of spectra into different test sets was done randomly. To create the "analogues test set, the mass spectra were first grouped when they were annotated with the same 2D structure. Subsequently, a random selection was made from the unique 2D structures to create the test sets; this was done to make sure that this test set was structure-disjoint . For the "exact match test set"again spectra were first grouped on 2D structure and for each 2D structure containing multiple spectra one spectrum was randomly selected for the test set. This was done to have a test set with at least one exact match in the reference library, making it possible to test the performance of predicting exact matches.
Blinding	A validation set was used during the training and optimization of hyperparameters of the model used. This validation set was also used to determine the best method for comparing performance, which was used in the 20-fold cross-validation. When analyzing the case studies, the results were not analyzed blinded. However, these results were not used to prove that MS2Query performs better than other standards in the field but instead were used to illustrate the variance in performance on different sample types and to illustrate examples on real data. During data collection no blinding to group allocation could be performed, since no comparison between groups were made, the case studies were only used to determine which and how many metabolites could be annotated.

Reporting for specific materials, systems and methods

We require information from authors about some types of materials, experimental systems and methods used in many studies. Here, indicate whether each material, system or method listed is relevant to your study. If you are not sure if a list item applies to your research, read the appropriate section before selecting a response.

Materials & experimental systems

n/a	Involved in the study
☒	☐ Antibodies
☒	☐ Eukaryotic cell lines
☒	☐ Palaeontology and archaeology
☒	☐ Animals and other organisms
☒	☐ Clinical data
☒	☐ Dual use research of concern

Methods

n/a	Involved in the study
☒	☐ ChIP-seq
☒	☐ Flow cytometry
☒	☐ MRI-based neuroimaging
